# Supplementary material for: The Small RNA Universe of Capitella teleta
Source: Front Mol Biosci. 2022 Feb 25;9:802814. doi: 10.3389/fmolb.2022.802814 (PMC8915122; doi:10.3389/fmolb.2022.802814)
Supplement: Supplementary file 1 [file DataSheet1.ZIP › Supplement/known/CAPTEscaffold_12_1220.pdf]

Provisional ID	:	CAPTEscaffold_12_1220
Score total	:	69.7
Score for star read(s)	:	3.9
Score for read counts	:	62.8
Score for mfe	:	1.9
Score for randfold	:	1.6
Score for cons. seed	:	-0.6
Total read count	:	135
Mature read count	:	121
Loop read count	:	0
Star read count	:	14

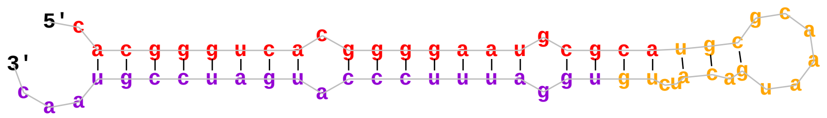

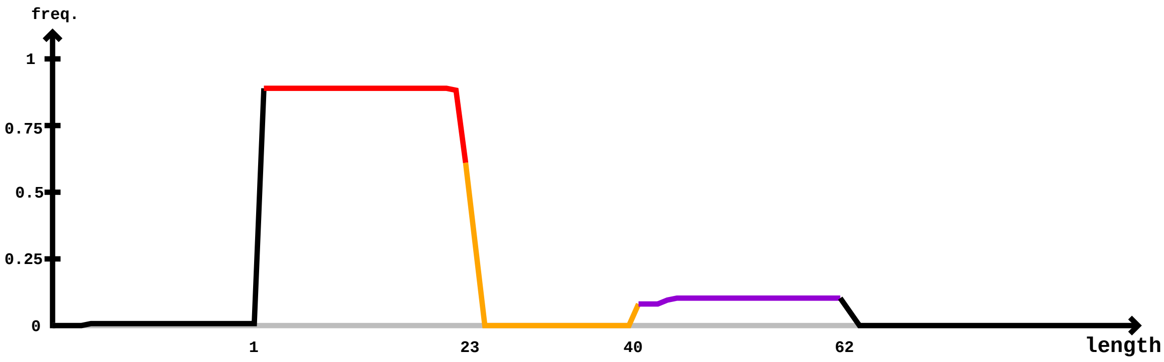

Mature Star

```
5'- caacagaggcacggcacaggcacgggucacggggaaugcgcaugcgcaaaugacaucuguggauuucccaugauccguaacugcguccugcucaaugucguuugcugaacu  -3'  obs
    caacagaggcacggcacaggcacgggucacggggaaugcgcaugcgcaaaugacaucuguggauuucccaugauccguaacugcguccugcucaaugucguuugcugaacu        exp
    caacagaggcacggcacaggcacgggucacggggaaugcgcaugcgcaaaugacaucuguggauuucccaugauccguaacugcguccugcucaaugucguuugcugaacu        known
    ..(((..((((.(((.(((..((((((((.(((((((.((((((......).))..)))).))))))).))))))))..))).))).))))...)))............   reads   mm   sample

    ..acagaggcacggcacagg...........................................................................................   1   0   seq
    ....................cacgggucacggggaaugcg.......................................................................   1   0   seq
    ....................cacgggucacggggaaugcgc......................................................................   37  0   seq
    ....................cacgggucacggggaaugcgca.....................................................................   82  0   seq
    ....................cacgggucacAgggaaugcgca.....................................................................   1   1   seq
    ...........................................................uggauuucccaugauccguaac..............................   10  0   seq
    ...........................................................uggauuucccaugauccguaacu.............................   1   0   seq
    ..............................................................auuucccaugauccguaac..............................   2   0   seq
    ...............................................................uuucccaugauccguaac..............................   1   0   seq
```
